# Effect of *brachytic2* mutation in corn silage on performance and total-tract digestibility by lactating Holstein dairy cows

**DOI:** 10.3168/jdsc.2025-0800

**Published:** 2025-08-13

**Authors:** E. Sarmikasoglou, D. Hammer, T. Dietz, M.J. VandeHaar

**Affiliations:** 1 Department of Animal Science, Michigan State University, East Lansing, MI 48824; 2 Bayer Crop Science, Chesterfield, MO 63141

## Abstract

Short-stature corn hybrids carrying the *brachytic2* (*br2*) mutation have greater standability and have grain yields similar to conventional tall hybrids; thus, they are promising for grain production. Our objective was to determine if short hybrids would also be valuable as sources of silage for lactating cows. We compared corn silages (CS) from 3 novel short hybrids (SH-1,2,3) to CS from tall (Tall) and *bm3* (BMR) hybrids for their effects on DMI, milk production, and total-tract nutrient digestibility. Twenty primiparous and 20 multiparous Holstein cows (150 ± 42 DIM, 35 ± 6.4 kg of milk/d, 674 ± 103 kg of BW; mean ± SD) were enrolled in an incomplete Latin square design with three 21-d experimental periods, balanced for carryover effects. Cows were fed diets with a blend of all CS for a 14-d preliminary period, and then experimental diets for 63 d. Experimental diets contained CS from Tall, BMR, SH1, SH2, or SH3, along with alfalfa silage, soybean meal, soybean hulls, ground corn, and a premix of protein, minerals, and vitamins. All diets were formulated for 18% CP and 24% forage NDF (forNDF) and fed as TMR. Data were analyzed using PROC GLIMMIX of SAS 9.4. Preplanned contrasts were Tall versus Short (Tall vs. SH-1,2,3) and BMR versus Short (BMR vs. SH-1,2,3). Compared with cows fed the diet with Tall, cows fed Short CS produced more milk, protein, and lactose, but their milk had a lower fat percentage, and ECM yield was not different. Compared with cows fed BMR CS, cows fed Short CS had greater DMI and yields of ECM, milk, protein, and lactose, but lower fat percentage, ECM/DMI, and BCS gain. Apparent total-tract digestibilities of OM, starch, and CP, but not NDF, were lower in cows fed BMR CS than those fed short hybrids. We conclude that short corn hybrids could be valuable sources of silage on dairy farms. Further studies in other growing environmental conditions seem warranted.

## Highlights

• Silage from *br2* corn hybrids improved milk and protein yield compared with tall corn.
• Silage from *br2* corn hybrids increased dry matter intake (DMI) and ECM yield compared with BMR silage.
• Cows fed *br2* silages had greater organic matter, crude protein, and starch digestibilities than cows fed BMR.
• NDF digestibility was similar between cows fed BMR and the *br2* corn silages.
• Feed efficiency was lower for cows fed corn silage from *br2* hybrids than BMR.

## Graphical Abstract

**Figure:**
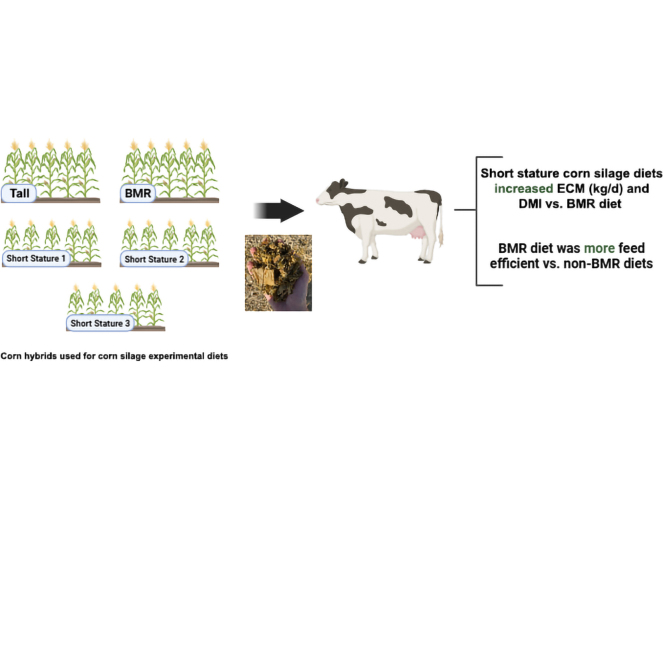
**Summary:** Short-stature corn hybrids that carry the *brachytic2* (*br2*) mutation have increased plant standability compared with conventional tall and brown midrib (BMR) hybrids but similar dry matter yields to tall hybrids. Diets containing silage from 3 *br2* hybrids were compared with diets containing silage from tall and BMR hybrids for their effects on cows in mid lactation when nutrient composition was similar. Cows fed diets with silage from the short hybrids produced as much or more energy-corrected milk (ECM) as cows fed diets with silage from tall or BMR hybrids. Intake was greater and feed efficiency was lower in cows fed short hybrids than in those fed BMR. We conclude that short-stature corn hybrids could be valuable sources of silage to improve production and profits on dairy farms. Created in BioRender.

Corn silage (**CS**) is a common forage source in dairy cattle diets due to its energy value, fiber content, and high yielding properties. Typically, CS is composed of 26% to 39% starch, and 36% to 46% NDF (DM basis; NASEM, 2021). By increasing starch and NDF digestibility, the feeding value of CS improves and milk production can be enhanced (Oba and Allen, 1999; Ferraretto and Shaver, 2012). The NDF digestibility is limited by the formation of cross-linkages between lignin and hemicellulose, resulting in a physical barrier to rumen degradation (Moore and Jung, 2001; Jung et al., 2012). Several strategies, including altered ensiling time (Hoffman et al., 2011), use of fibrolytic enzymes (Beauchemin et al., 2003), bacterial inoculants (Muck et al., 2018), and chemical additives (Auerbach et al., 2012), as well as genetic modification (Jung et al., 2012), have aimed to improve CS NDF digestibility. Utilizing CS hybrids with lower lignin content, such as *bm3* brown midrib (**BMR**) hybrids, has been identified as an efficient method to increase NDF digestibility. The inclusion of CS from *bm3* corn increases DMI and improves milk production compared with CS from conventional corn hybrids (Oba and Allen, 2000; Ebling and Kung, 2004). However, *bm3* corn typically has lower DM yields and poorer standability compared with conventional corn (Sattler et al., 2010; Wallau et al., 2022).

Corn hybrids carrying the short-stature (**SH**) mutation *brachytic2* (*br2*) exhibit shorter internodes, more erect leaves, and reduced plant height than conventional tall hybrids (Pilu et al., 2007; Barten et al., 2022). Most importantly, corn hybrids with *br2* have greater standability and decreased losses in productivity during windstorms (Barten et al., 2022). Previously, Byers et al. (1965) evaluated the feeding value of short hybrids as a silage source for dairy cattle diets and found no effects on DMI, FCM, or BW change; however, whether those short hybrids contained the *br2* mutation is not known. To our knowledge, no studies have been published in lactating cows on the feeding potential of CS with *br2* genetics, but one unpublished study found no effects on DMI, ECM, or BW (A. Catellani, C. Mastroeni, L. F. Ferraretto, A. Fiorini, E. Trevisi, J. L. Pellet, E. Badalotti, M. Battisti, and A. Gallo [Università Cattolica del Sacro Cuore, Piacenza, Italy]; personal communication). Therefore, the objective of this study was to determine if diets containing CS from 3 novel hybrids with the *br2* mutation would alter DMI, milk production, and apparent total-tract nutrient digestibility of Holstein cows compared with diets containing CS from conventional tall and *bm3* corn hybrids.

All experimental procedures were approved by the Michigan State University Institutional Animal Care and Use Committee. Tall (DKC59–07RIB, DEKALB, Bayer Crop Science, Leverkusen, Germany), BMR (P0956AMX, Pioneer, Pioneer Hi-Bred International Inc., Johnston, IA), and 3 SH (**SH1**, **SH2**, and **SH3**, Bayer Crop Science, Leverkusen, Germany) corn hybrids were each planted at 86,500 seeds/ha in adjacent field plots of 2.19 ha each at the Michigan State University Dairy Cattle Teaching and Research Center (East Lansing, MI) on May 15, 2023, and grown under the same tillage, fertilizer application, and weed control practices (Explorer, Syngenta, Basel, Switzerland). The relative maturities (**RM**) of Tall, BMR, SH1, SH2, and SH3 were 109, 109, 112, 111, and 108 d RM, respectively. All hybrids were clipped near ground level and chopped at 34% DM on September 21 and 22, 2023, with theoretical length of cut at 1.60 cm and using a kernel processor. All hybrids were treated with 2 g/t (400,000 cfu *Lactobacillus buchneri* and 100,000 *Pediococcus pentosaceus* per gram of forage) of MAGNIVA Titanium inoculant (Vita Plus Corporation, Madison, WI) at harvest, ensiled in separate silo bags, and allowed to ferment for approximately 100 d before feeding. Yields of DM harvested were 19, 17, 21, 17, and 19 t/ha for Tall, BMR, SH1, SH2, and SH3, respectively. About 2 wk before the start of the study, samples were collected from the front and at two 6-m intervals on the sides of each silo bag and analyzed for nutrient composition by Cumberland Valley Analytical Services (Waynesboro, PA). The in vitro NDF digestibility (**IVNDFD**; % NDF) after 30 h of incubation of Tall, BMR, and SH-1,2,3 was 53% ± 0.8%, 63% ± 1.1%, 60% ± 1.3%, 57% ± 0.6%, and 57% ± 1.1%, respectively. The nutrient composition of CS is presented in Table 1 footnotes.

**Table 1:** Ingredient and nutrient composition of experimental diets

Item	Treatment^1^					
	Tall	BMR	SH1	SH2	SH3	PRE
% DM						
Tall CS^2^	55.0	—	—	—	—	11.0
Brown midrib CS^3^	—	58.0	—	—	—	11.6
Short stature 1 CS^4^	—	—	54.0	—	—	10.8
Short stature 2 CS^5^	—	—	—	60.0	—	12.0
Short stature 3 CS^6^	—	—	—	—	59.0	11.8
Alfalfa haylage	10.0	10.0	10.0	10.0	10.0	10.0
Soybean meal	14.8	14.5	13.7	14.9	14.6	14.5
Soybean hulls	5.60	4.60	3.50	3.80	4.50	4.40
Corn, dry ground	3.80	2.10	8.00	0.50	1.10	3.10
Concentrate mix^7^	8.00	8.00	8.00	8.00	8.00	8.00
Mineral and vitamin mix^8^	2.80	2.80	2.80	2.80	2.80	2.80
Nutrient composition						
NDF (% of DM)	29.6	29.5	29.3	28.2	28.6	29.0
forNDF (% of DM)	23.9	24.6	24.5	23.9	23.8	24.2
CP (% of DM)	18.1	18.1	18.1	18.3	18.1	18.1
Starch (% of DM)	25.2	25.0	26.6	26.5	25.9	25.9
Fatty acids (% of DM)	2.20	2.10	2.20	2.30	2.10	2.20
RUP (% of CP)	34.0	34.0	34.0	34.0	34.0	34.0
RDP (% of DM)	11.9	11.9	11.9	12.1	11.9	12.0
NEL (Mcal/kg)	1.57	1.59	1.64	1.61	1.63	1.61

1 Tall = tall corn silage diet; BMR = brown midrib corn silage diet; SH1 = short-stature corn silage 1 diet; SH2 = short-stature corn silage 2 diet; SH3 = short-stature corn silage 3 diet; PRE = covariate period diet.

2 Tall corn silage (34.5 ± 0.71% DM; mean ± SD) nutrient composition used to formulate Tall diet (% DM): 35.9% ± 0.66% NDF, 22.0% ± 0.86% ADF, 2.74% ± 0.14% lignin, 7.10% ± 0.22% CP, and 40.4% ± 1.62% starch.

3 Brown midrib corn silage (34.9 ± 0.90% DM) nutrient composition used to formulate BMR diet (% DM): 34.0% ± 0.12% NDF, 20.8% ± 0.31% ADF, 1.96% ± 0.15% lignin, 7.67% ± 0.12% CP, and 40.0% ± 1.55% starch.

4 Short stature corn silage 1 (33.0% ± 0.43% DM) nutrient composition used to formulate SH1 diet (% DM): 36.5% ± 0.85% NDF, 21.9% ± 0.29% ADF, 2.32% ± 0.09% lignin, 8.13% ± 0.19% CP, and 35.2% ± 0.49% starch.

5 Short stature corn silage 2 (34.4 ± 0.43% DM) nutrient composition used to formulate SH2 diet (% DM): 32.8% ± 0.78% NDF, 19.5% ± 0.78% ADF, 2.34% ± 0.09% lignin, 7.40% ± 0.08% CP, 40.1% ± 0.37% starch.

6 Short stature corn silage 3 (34.1% ± 1.30% DM) nutrient composition used to formulate SH3 diet (% DM): 33.4% ± 0.95% NDF, 19.8% ± 0.64% ADF, 2.27% ± 0.16% lignin, 7.67% ± 0.22% CP, 40.5% ± 1.04% starch.

7 Concentrate mix contained (% as fed) 40.82% Amino Plus (Ag Processing Inc.), 17.69% corn grain, 14.64% sodium sesquinate, 11.97% Spectrum AgriBlue (Perdue AgriBusiness), 11.9% calcium carbonate, 2.45% urea, and 0.53% Smartamine M (Adisseo).

8 Mineral and vitamin supplement mix contained (% as fed) 27.84% soybean meal, 21.65% DCAD, 11.87% calcium phosphate, 7.56% corn grain, 7.26% salt, 0.47% tallow, and trace minerals and vitamins to meet NASEM (2021) requirements.

The feeding study was conducted during the months of January to April of 2024 at Michigan State University Dairy Cattle Teaching and Research Center (East Lansing, MI). Twenty primiparous (145 ± 43 DIM, 32 ± 4.5 kg of milk/d, 611 ± 83 kg of BW) and 20 multiparous Holstein cows (154 ± 41 DIM, 39 ± 5.65 kg of milk/d, 740 ± 76 kg of BW) were enrolled in an incomplete Latin square design with three 21-d periods, balanced for carryover effects. Cows were blocked by parity, as primiparous or multiparous, and randomly assigned to one of the treatment sequences. Cows were housed in individual tiestalls and milked thrice daily. Water was available ad libitum. Feed was offered once daily as a TMR at 0800 h at 115% of expected intake and was continuously available until the next morning at 0700 h, except during milk and exercising. Due to clinical mastitis, one cow was removed from the study and her data were excluded from statistical analysis.

Cows were fed a preliminary diet for 14 d and then experimental diets for 63 d total (Table 1). Experimental diets contained one of the 5 CS, along with alfalfa silage, soybean meal, soybean hulls, ground corn, and a premix of protein, minerals, and vitamins (Table 1). All diets were formulated based on the nutrient composition of feed ingredients before the start of the study to provide 18% CP, 24% forage NDF (**forNDF**), and 26% starch (DM basis) and to meet mineral and vitamin requirements according to NASEM (2021). Feed analyses were conducted during the study, and the nutrient composition presented in Table 1 refers to the average of these.

Milk yield was recorded daily, and milk samples were collected at each milking for 4 consecutive days on the last week of each period. Samples were stored with preservative (Bronolab W-II liquid, Advanced Instruments) at 4°C until analysis. Individual milk samples were analyzed by CentralStar Cooperative Inc. for fat, true protein, lactose, MUN, and SCC concentrations by mid-infrared spectroscopy (AOAC, 1990) using a Bentley FTS instrument. Milk and component yields for each milking were summed for a daily total and to calculate the ECM and milk component yields. The ECM was calculated as

$$\mathrm{ECM}=\frac{\left(\mathrm{NE}L_{\mathrm{NASEM},\;2021}\times \mathrm{kg}\;\mathrm{of}\;\mathrm{milk}\right)}{0.7},$$

where milk energy output was estimated using equation 3–14b from NASEM (2021):

$$\begin{array}{c} \mathrm{NEL}\;\left(\mathrm{Mcal}/\mathrm{kg}\right)=9.29\times \frac{\mathrm{kg}\;\mathrm{of}\mathrm{fat}}{\mathrm{kg}\;\mathrm{of}\mathrm{milk}}+5.85\times \frac{\mathrm{kg}\;\mathrm{of}\mathrm{true}\;\mathrm{protein}}{\mathrm{kg}\;\mathrm{of}\mathrm{milk}}\\ \;+3.95\times \frac{\mathrm{kg}\;\mathrm{of}\mathrm{lactose}}{\mathrm{kg}\;\mathrm{of}\mathrm{milk}}. \end{array}$$

Cows were weighed 3 consecutive days in the last week of each period immediately following afternoon milkings. Body condition score for each cow was measured as the average score of 3 trained investigators using a 5-point scale on the last day of each period.

Samples of all diet ingredients and TMR (∼0.5 kg) were collected once weekly and stored at −20°C until composited by period and dried; these values were used for calculating final diet composition (Table 1). Apparent total-tract digestibilities were determined on 39 cows over the last 5 d of period 2. Samples of feed ingredients (∼0.5 kg) and orts (12.5% of remaining feed) for individual cows were collected daily and composited for each cow. Feces (∼400 g) were collected every 15 h over the 5 d, resulting in 8 samples/cow representing every 3 h of a day. Feces were stored at −20°C until dried and composited on an equal DM basis for each cow. Diet ingredients, orts, and fecal samples were dried at 55°C for 72 h in a forced-air oven to determine DM. Dried samples were ground with a Wiley mill (5-mm screen; Arthur H. Thomas). Samples of diet ingredients, orts, and feces were analyzed by Cumberland Valley Analytical Services (Waynesboro, PA) for CP (AOAC International, 2000), starch (Hall, 2009), and ash (method 942.05; AOAC International, 2000) modified to ash a 1.50-g sample for 4 h. The NDF and undegradable NDF were determined according to Mertens et al. (2002) and Goering and Van Soest (1970), respectively. Undegradable NDF, estimated as NDF residue after 240 h of in vitro fermentation, was used as an internal marker to predict fecal output (Cochran et al., 1986). Apparent total-tract digestibilities of nutrients were calculated as in Daniel et al. (2020).

Normality of residuals and homogeneity of variance were examined for each continuous dependent variable. All statistical analyses were performed using the GLIMMIX procedure of SAS 9.4 (SAS Institute Inc., Cary, NC). The statistical model to analyze responses only from wk 3 of each period included the fixed effects of treatment; parity group (primiparous vs. multiparous); period; the interaction between treatment and parity; the interaction between treatment and period; the interaction between parity and period; the interaction between treatment, parity, and period; and the DIM of each cow on d 1 of the start of the experiment. Preplanned contrasts were (1) Tall vs. Short (Tall vs. SH-1,2,3) and (2) BMR vs. Short (BMR vs. SH-1,2,3). Significance was declared at *P* ≤ 0.05, and tendency was declared at 0.05 < *P* ≤ 0.10.

On average, cows produced 32.8 kg of milk daily with 4.37% fat, 3.47% true protein, 4.86% lactose, and 5.41 mg of MUN/dL (Table 2). Cows fed short CS had greater yields of milk (*P* = 0.02), protein (*P* < 0.01), lactose (*P* < 0.01), but lower milk fat percentage (*P* < 0.01) compared with cows fed Tall. Cows fed the short CS had greater DMI (*P* < 0.01) and yields of milk (*P* < 0.01), ECM (*P* = 0.01), protein (*P* < 0.01), and lactose (*P* < 0.01), but lower milk fat percentage (*P* < 0.01), ECM/DMI (*P* < 0.01), and BCS gain (*P* < 0.01) than did cows fed BMR.

**Table 2:** Effects of corn silage hybrids on milk production and BW

Variable	Treatment^1^					SEM	*P*-value^2^	
	Tall	BMR	SH1	SH2	SH3		Tall vs. Short	BMR vs. Short
DMI (kg)	26.2	24.5	27.8	25.8	25.9	0.58	0.35	<0.01
Milk (kg/d)	32.5	32.0	33.7	33.2	32.7	0.74	0.02	<0.01
ECM^3^ (kg/d)	37.0	36.5	38.7	37.3	36.9	0.79	0.11	0.01
Fat (%)	4.43	4.45	4.34	4.30	4.33	0.07	<0.01	<0.01
Fat (kg/d)	1.41	1.40	1.46	1.41	1.41	0.03	0.39	0.14
Protein (%)	3.48	3.46	3.52	3.43	3.46	0.04	0.61	0.64
Protein (kg/d)	1.11	1.10	1.19	1.14	1.13	0.02	<0.01	<0.01
Lactose (%)	4.86	4.85	4.88	4.86	4.85	0.02	0.92	0.22
Lactose (kg/d)	1.56	1.55	1.65	1.60	1.59	0.04	<0.01	<0.01
MUN (mg/dL)	5.32	5.36	5.38	5.56	5.45	0.17	0.11	0.25
SCC^4^ (× 1,000 cells/mL)	10.2	8.46	9.35	9.48	8.62	0.80	0.22	0.38
ECM/DMI	1.44	1.51	1.39	1.45	1.44	0.02	0.60	<0.01
Captured energy/feed energy intake^5^	0.68	0.73	0.67	0.68	0.74	0.03	0.64	0.17
BW (kg)	691	690	693	690	696	12.9	0.34	0.23
BW gain (kg/d)	0.93	0.87	0.93	0.80	1.12	0.14	0.85	0.48
BCS	3.25	3.38	3.31	3.23	3.18	0.07	0.88	0.01
BCS change (U/21 d)	−0.0047	0.0005	−0.0034	−0.0050	−0.0064	0.0023	0.91	0.02

1 Tall = tall corn silage diet; BMR = brown midrib corn silage diet; SH1 = short-stature corn silage 1 diet; SH2 = short-stature corn silage 2 diet; SH3 = short-stature corn silage 3 diet.

2 Tall vs. Short = Tall vs. SH-1,2,3; BMR vs. Short = BMR vs. SH-1,2,3.

3 ECM = (NEL, equation 3–14b, NASEM, 2021, × kg of milk)/0.7.

4 Due to nonnormality, SCC was log-transformed for analysis. Means were back-transformed for reporting. SEM were calculated by using the delta method for SEM estimations.

5 Captured energy/feed energy intake = NEL + body energy change.

Total-tract digestibilities of OM (*P* < 0.01), starch (*P* < 0.01), and CP (*P* < 0.01) of cows fed CS from short hybrids were less than those of cows fed CS from BMR (Table 3) and similar to those of cows fed Tall. Surprisingly, digestibility of NDF was similar for cows regardless of hybrid.

**Table 3:** Effects of corn silage hybrids on apparent total-tract digestibility of nutrients

Variable	Treatment^1^					SEM	*P*-value^2^	
	Tall	BMR	SH1	SH2	SH3		Tall vs. Short	BMR vs. Short
OM digestibility (%)	62.4	52.2	61.3	58.0	60.3	1.65	0.19	<0.01
NDF digestibility (%)	47.2	45.4	45.2	46.1	44.2	1.16	0.15	0.88
Starch digestibility (%)	99.0	98.1	99.1	99.3	99.4	0.17	0.12	<0.01
CP digestibility (%)	71.0	67.2	71.1	72.1	70.0	0.74	0.97	<0.01

1 Tall = tall corn silage diet; BMR = brown midrib corn silage diet; SH1 = short-stature corn silage 1 diet; SH2 = short-stature corn silage 2 diet; SH3 = short-stature corn silage 3 diet.

2 Tall vs. Short = Tall vs. SH-1,2,3; BMR vs. Short = BMR vs. SH-1,2,3.

In previous studies, cows in early lactation fed BMR CS in high forNDF diets had greater DMI, and milk production, with no change in feed efficiency compared with those fed conventional CS diets (Oba and Allen, 2000; Qiu et al., 2003; Ferraretto et al., 2015). In our study, cows in mid lactation fed the BMR-based diet exhibited lower intake and milk yield, but greater feed efficiency compared with those fed diets containing short CS or Tall. Cows in the early postpartum period usually are in negative energy balance, and gastric fill is considered as the main factor limiting intake (Allen, 1996). Cows in our study were in mid rather than early lactation, which might explain the lack of a production response to the BMR CS in this study. However, we fed diets with greater NDF (∼29% of DM) and forNDF (24% of DM) and less starch (26% of DM) than is optimal for mid-lactation cows, and when we switched cows to our preliminary diet from their normal herd diet, their DMI and milk yield dropped by 3.6 and 4.8 kg/d, respectively. Thus, based on the greater 30-h-IVNDFD of BMR compared with other hybrids, we expected the BMR diet to have increased DMI and milk production in this study compared with what we observed. Possible explanations for the lack of benefit of BMR corn in our study could be the lactation stage of cows, mycotoxins (not tested), or the unusual weather of 2023 (a drought year in MI).

The partial replacement of conventional CS with forages containing *bm3* and dwarf genetics has been previously investigated in drought-tolerant species of sorghum (*Sorghum bicolor*) and pearl millet (*Pennisetum glaucum*) with promising results on performance. Replacing CS with silage from dwarf *bm3* sorghum at 10% of the total diet DM increased milk fat content and maintained ECM in cows when NDF and starch contents were kept constant at 34% and 21% of DM, respectively (Harper et al., 2017). In addition, replacement of traditional CS by pearl millet silage at 10% of the total diet DM maintained high milk production in mid-lactation cows (Harper et al., 2018). However, to our knowledge, this is the first study reporting the feeding value of CS with *br2* genetics, a mutation gained through natural selective breeding, on milk production.

We conclude that corn hybrids with the *br2* mutation produce CS with a feeding value for dairy cows that is at least as good and likely better than conventional (Tall) or BMR corn varieties. Moreover, for 2 of the SH varieties, DM yields/ha were as great or better than the Tall hybrid. Therefore, based on their standability, DM yield, and feeding value for milk production, we conclude that short-stature corn hybrids could be valuable sources of silage to improve production and profits on dairy farms and deserve further study.
